# Legacy Effects in Buds and Leaves of European Beech Saplings (*Fagus sylvatica*) after Severe Drought

**DOI:** 10.3390/plants12030568

**Published:** 2023-01-26

**Authors:** Frank M. Thomas, Lena Schunck, Alexis Zisakos

**Affiliations:** Faculty of Spatial and Environmental Sciences, Geobotany, University of Trier, Behringstraße 21, 54296 Trier, Germany

**Keywords:** bud formation, climate change, deciduous tree, drought damage, foliar nitrogen, isotopic signature, plasticity, resilience, triphenyltetrazolium chloride

## Abstract

Against the background of climate change, we studied the effects of a severe summer drought on buds of European beech (*Fagus sylvatica* L.) saplings and on leaves formed during the subsequent spring in trees attributed to different drought-damage classes. For the first time, we combined assessments of the vitality (assessed through histochemical staining), mass and stable carbon isotope ratios (δ^13^C) of buds from drought-stressed woody plants with morphological and physiological variables of leaves that have emerged from the same plants and crown parts. The number, individual mass and vitality of the buds decreased and δ^13^C increased with increasing drought-induced damage. Bud mass, vitality and δ^13^C were significantly intercorrelated. The δ^13^C of the buds was imprinted on the leaves formed in the subsequent spring, but individual leaf mass, leaf size and specific leaf area were not significantly different among damage classes. Vitality and δ^13^C of the buds are suitable indicators of the extent of preceding drought impact. Bud vitality may be used as a simple means of screening saplings for the flushing capability in the subsequent spring. European beech saplings are susceptible, but—due to interindividual differences—are resilient, to a certain extent, to a singular severe drought stress.

## 1. Introduction

In the course of climate change, increased frequency and intensity of hot and dry summer periods in recent years have caused damage to forests and forest trees. Such damage has also affected trees and stands of the European beech (*Fagus sylvatica* L.) [1,2,3,4,5,6,7,8], the ecologically and economically most important deciduous forest tree species in Central Europe [9]. Effects of climate change and weather extremes on the beech have sparked a discussion on the species’ suitability for the future silviculture in warmer regions of Central Europe [10,11,12]. In a precipitation exclusion experiment on seedlings of East-Asian trees, including broad-leaved species [13], which was conducted to create a scientific foundation of species selection for afforestation programmes, prolonged drought resulted in morphological alterations, including reductions in growth and specific leaf area (SLA), as well as in physiological responses such as a decrease in the leaf water potential and net photosynthesis and an increase in the ratio of stable carbon isotopes (δ^13^C). In principle, drought legacy effects on growth, water relations, gas exchange and concentrations of nitrogen (N) and non-structural carbohydrates (NSC) have been widely studied in mature broadleaf–deciduous and coniferous trees up until the most recent past [14,15,16,17,18,19,20,21], but investigations of drought effects on the buds formed in late summer and on the leaves emerged from buds in the subsequent spring are very rare. At least, it has been found that the number of flushes in saplings of Central-European oak species was significantly lower in the second and third year of drought stress [22]. In a previous study [23], the mass of individual buds, but not the bud number per tree was significantly reduced in two-year-old seedlings of the European beech. However, the vitality of beech buds as a measure of flushing capability and the δ^13^C ratios in buds as an indicator of drought stress [24] has not been investigated so far. 

In the present study, we took advantage of a warm and extremely dry spring and summer that enabled us to investigate the effects of severe drought on buds and its legacy effects on the subsequent year’s leaves in European beech saplings cultivated in an open space. In the earlier study [23], foliar δ^13^C values of bulk leaf material sampled in the summer and early autumn did not differ between severely drought-stressed and control trees, most probably because the bulk of the leaves had been generated before the onset of drought stress. In the European beech, however, a fixed (determinate) shoot growth pattern prevails, in which the winter buds contain primordia of all leaves that will expand in the subsequent spring [25]; and the δ^13^C of NSC, which would be used for leaf formation in the next growing season, was significantly increased in European beech saplings upon severe drought stress [26]. On the basis of all those previous findings, we hypothesised that (1) not only the number and the individual mass of the buds, but also their vitality, assessed through histochemical staining and taken as a measure of flushing capability, would be reduced in severely injured plants; that bud mass and vitality are correlated; and that the δ^13^C values of buds should be higher in trees of higher damage classes. We also expected that (2) leaves formed from ^13^C-enriched buds of severely drought-injured trees should not only exhibit a lower mass and smaller size, but also an increased δ^13^C ratio, resulting in a significant correlation between the δ^13^C signatures of buds and leaves. Accordingly, we hypothesised (3) significant correlations between bud vitality and bud δ^13^C, as well as between the masses of the buds and their δ^13^C ratios. Finally, (4) leaf mass should correlate significantly with bud mass. If the histochemically determined vitality and the mass and the δ^13^C signature of the buds would prove to be interrelated, they may also be used interchangeably in the future to assess the flushing probability and, thus, the growth potential of tree seedlings and saplings not only in nurseries but also in forest stands to predict recruitment success.

## 2. Materials and Methods

### 2.1. Plant Cultivation, Drought Damage Classification and Sampling

**Figure 1:**
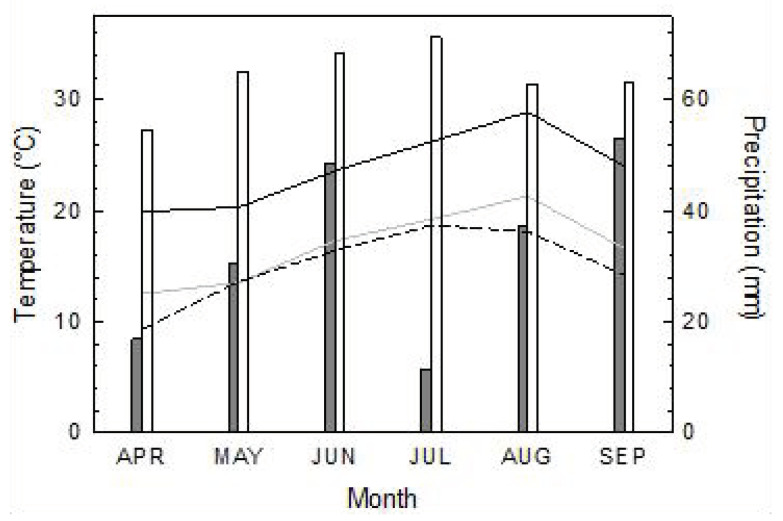
Monthly averages of maximum (solid black line) and mean temperature (grey line) and monthly sums of precipitation (grey bars) in spring and summer 2020, and long-term monthly averages (1981–2010) of temperature (dashed line) and precipitation (white bars) for comparison.

In March 2020, 40 three-year-old saplings of European beech (*Fagus sylvatica* L.) of the height class 60–100 cm had been obtained from a commercial tree nursery. They were kept in standard garden soil in 14-L plastic pots and placed in an open space outside the greenhouse (49°44′50″ N; 006°41′03″ E; 249 m a.s.l.) of Trier University’s Geobotany department. There, the trees were exposed to the ambient weather conditions that were characterised by a warm–dry period from April to September (Figure 1): the monthly mean temperature was above the long-term average except for May, and the monthly precipitation was below average during the entire spring and summer (weather data originated from the weather station Trier-Petrisberg of the German Meteorological Service (Deutscher Wetterdienst, DWD [27]; 49°44′52″ N; 006°39′30″ E; 261 m a.s.l., approximately 2 km linear distance from the greenhouse) and were retrieved from [28]). July and August were particularly dry and even met the definition of an arid period according to Walter’s type of climate diagram [29] when the precipitation figure is below the line of the average temperature (Figure 1). In September, 97% of the month’s precipitation, which equalled a quarter of the entire rainfall from April to September, fell in the month’s last week. After September, the plants were watered just enough to prevent their death. In March 2021, another twenty beech trees (height class 80–120 cm, to compensate for the growth increment of the saplings bought in the preceding year), were purchased from the same tree nursery and were then used as unstressed control (C) trees. 

In autumn 2020, the height and the stem diameter of the trees (5 cm above the soil surface; average of two measurements perpendicular to each other) were measured and their leaf buds were counted. All trees were attributed to classes of damage caused by drought stress based on the extent of leaf loss, as well as leaf and stem discolouration. By combining the extents of damage and following the conventional classification scheme of the Level I monitoring programme for the crown condition of European forests (e.g., [30]), we assigned the trees to four damage classes (DC): DC1 (0–25% damage), DC2 (>25–60% damage), DC3 (>60–90% damage) and DC4 (>90% damage). The number of trees in each damage class is given in Table 1. For further investigations, four trees were randomly selected from each damage class. From each of those trees, two buds per crown part (upper, middle, and lower) and tree were harvested for further analyses. During winter, all plants were kept in the open space outside the greenhouse until the subsequent spring.

In May 2021, after complete leaf unfolding, five leaves per crown part were sampled from the trees selected in 2020 for bud analyses (four trees per DC) and from four randomly selected control trees. One tree each of DC2 and DC3 had to be replaced by another tree of the same damage class, as the original trees could not be identified anymore. In DC3, only one out of the four trees formed leaves in the upper crown part. All trees of DC4 did not leaf out anymore and had therefore to be excluded from further analyses. 

### 2.2. Analyses of Buds

After the fresh mass of each harvested bud had been determined, the buds were longitudinally cut into halves. One half was used for the “vitality” analyses and the other for the ratio of stable carbon isotopes (δ^13^C). 

The “vitality” test was conducted with the TTC (2,3,5-triphenyltetrazolium chloride) method, a histochemical staining approach that is widely employed for assessing tissue vitality, including organs of the European beech [31]. Here, we used the results of this test as a proxy of the flushing capability of the buds, as has been carried out in a previous study [32]. In healthy tissues, water-soluble TTC is reduced by electrons resulting from the mitochondrial respiratory chain to the red and insoluble 2,3,5-triphenyl formazan (TF) [33]. The intensity of the red colour is a measure of the tissue’s “vitality”.

We added 1% aqueous TTC solution to the bud halves that had been placed in Petri dishes. Subsequently, the buds in the dishes were vacuum infiltrated in an exsiccator for 30 min and then incubated for 3 h at 30 °C. The colouration of the cut surfaces of the buds was then used to assign them to the following vitality classes: bright red, red or purple surfaces—class 1 (high vitality and flushing capability); patchy to weakly red, but clearly visible colouration—class 2 (reduced vitality); weakly to brownish colouration—class 3 (sub-vital) [32]. For illustration, examples of the staining results are given in Figure S1 of the Supplementary Material.

The other halves of the buds (or buds from the same tree and crown position if the remaining material was insufficient) were cut with a scalpel in very fine pieces, frozen to −70 °C for 17 h and lyophilised for 24 h. Thereafter, 0.3 to 0.7 mg of bud material was weighed into tin capsules and analysed for δ^13^C with an IRMS Delta V™ isotope ratio mass spectrometer (Thermo Scientific, Bremen, Germany; two replicates per sample) with IAEA-CH-3 cellulose as a standard.

### 2.3. Analyses of Leaves

Upon harvest in May 2021, the size of each leaf was measured with a portable area meter (LI-3000A, LI-COR, Lincoln, NE, USA). Subsequently, the leaves were oven-dried for 24 h at 65 °C and weighed. From the leaf sizes and masses, the specific leaf area (SLA; m^2^ kg^−1^) was calculated. Until further analysis, the leaf samples were stored in an exsiccator.

For the measurement of the N concentration and δ^13^C, the same leaves that had been used for the SLA determination were pulverised in a swing mill grinder. The foliar N concentrations were measured in an element analyser. The δ^13^C ratios were determined in 2.0–2.5 mg of leaf material, as has been stated for the buds (see Section 2.2).

### 2.4. Statistical Analyses

Mean values ± 1 standard error (SE) are given if not stated otherwise. Statistical analyses were conducted with SigmaPlot for Windows Version 13.0, Build 13.0.0.83 (Systat Software, Inpixon, Palo Alto, CA, USA). Differences among the tree damage classes in tree height and stem diameter were tested with One-Way ANOVA, and in the number of buds, with the Kruskal–Wallis test, followed by Dunn’s method for multiple comparisons. We used Two-Way ANOVA, followed by pairwise comparisons according to Holm–Sidak, to test differences among the tree damage classes and crown parts (fixed factors) in the variables bud and leaf mass, leaf size, SLA, foliar N concentration and δ^13^C of buds and leaves. For the tests on the harvested buds, due to their limited number, we pooled all the buds per crown part across all trees per damage class to increase the statistical power of the tests (eight buds per crown part and damage class); whereas for the tests on leaves, we used mean values calculated separately for each tree and crown region (five leaves per tree and crown part). We calculated Spearman’s rank correlation coefficient to detect correlations between mass and vitality and between vitality and δ^13^C of the buds and used Pearson product-moment correlation to find relationships between mass and δ^13^C of the buds, between bud mass and leaf morphology and between bud and leaf δ^13^C.

## 3. Results

At the end of the 2020 growing season, the trees of the damage classes did not differ in height and stem diameter, but the extent of damage to the leaves and to the stems increased from DC1 to DC4 and the number of buds was significantly smaller in DC3 and DC4 than in DC1 (Table 1). Trees of the damage classes 3 and 4 also exhibited lower masses of the individual buds and smaller numbers of vital buds. In the buds of the DC3 and DC4 trees, the δ^13^C values were higher (less negative) than in the saplings of DC1. Across all damage classes, bud mass and δ^13^C differed significantly among crown parts (Table 2), but within damage classes, significant differences among the crown parts were restricted to DC3. There, the bud mass of the uppermost crown part and the δ^13^C ratios in the lowest crown part were the lowest. In DC2 to DC4, there was also a tendency for a lower number of vital and a higher number of sub-vital buds along the crowns from their lower to upper parts (Table 1).

**Figure 2:**
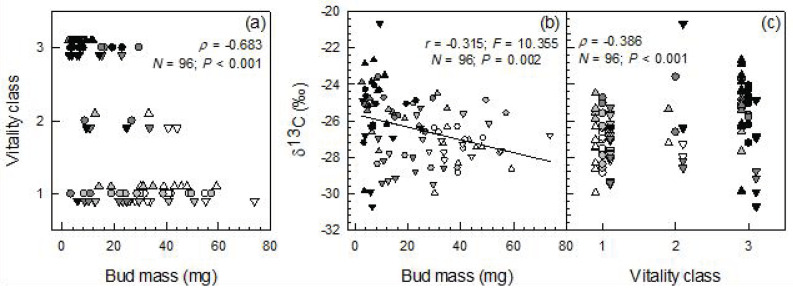
(**a**) Bud vitality classes vs. individual bud mass; (**b**) bud δ^13^C vs. individual bud mass; (**c**) bud δ^13^C vs. bud vitality in European beech saplings. The bud vitality classes ranged from 1, high vitality and flushing probability to 3, low vitality and flushing probability. Buds were harvested from different parts of the crown (triangles up, upper part; circles, middle part; triangles down, lower part) of saplings assigned to different drought damage classes (DC; white symbols = DC1, almost no damage; light grey symbols = DC2, moderate damage; dark grey symbols = DC3, severe damage; black symbols = DC4, very severe damage; cf. Section 2.1 and Table 1). *r*, Spearman correlation coefficient. To improve readability, the symbols of the different crown positions are slightly staggered around the respective vitality classes in panels (**a**,**c**).

**Figure 3:**
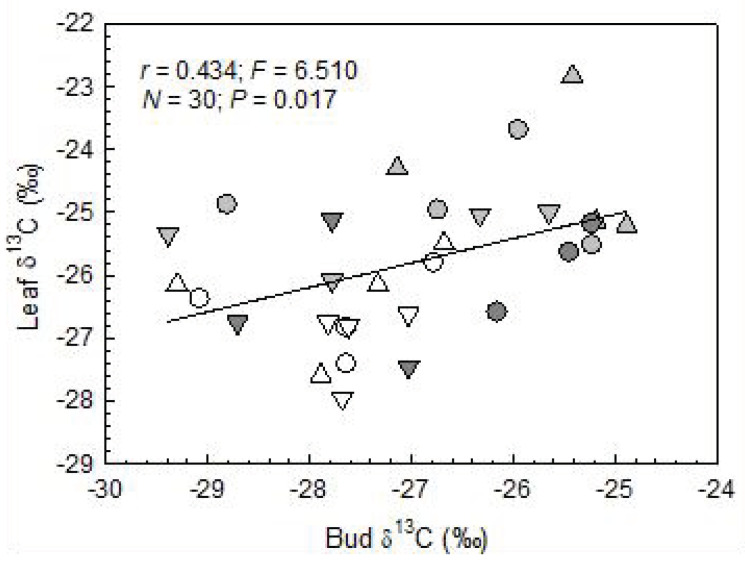
Leaf vs. bud δ^13^C in European beech saplings of different tree damage classes (DC; white symbols = DC1, almost no damage; light grey symbols = DC2, moderate damage; dark grey symbols = DC3, severe damage) and in different crown parts (triangles up, upper part; circles, middle part; triangles down, lower part).

Bud mass was significantly correlated with vitality and δ^13^C, and vitality was also significantly related to δ^13^C (Figure 2a–c). In the spring after the year of extreme summer drought, the trees of DC4 did not leaf out anymore. Likewise, buds did not unfold in the uppermost crown part in three out of the four DC3 plants. In the leaves that had been formed from the preceding year’s buds, no significant differences were found in dry mass, leaf size and SLA among damage classes (including control) (Table 3). However, the SLA tended to decrease from the lower to the upper crown region (significant differences in the control and DC2 and across all damage classes) (Table 2 and Table 3). Significant differences were found among the damage classes in the foliar δ^13^C ratios and N concentrations, with significantly higher (more positive) δ^13^C values in DC2 and significantly higher N concentrations in DC2 and DC3 compared to the other classes. The crown parts did not differ significantly in these variables (Table 2 and Table 3). The δ^13^C values of the leaves formed in spring 2021 were significantly and positively correlated with the δ^13^C ratios of the buds set in the preceding year at the same trees and crown regions (Figure 3). In contrast, the masses of the individual leaves (*r* = −0.003; *p* = 0.986), their sizes (*r* = −0.069; *p* = 0.703) and SLA (*r* = −0.139; *p* = 0.440) were not significantly related to the bud mass (*N* = 33 in these regressions).

## 4. Discussion

To our knowledge, this is the first study to combine assessments of the vitality, mass and δ^13^C ratios of buds from severely drought-stressed plants with morphological and physiological variables of leaves that have emerged from the same plants and crown parts. As a confirmation of our first hypothesis, the number, the individual mass and the vitality of the buds were significantly reduced in the severely injured plants of the higher damage classes and the δ^13^C signature was higher in the buds of these trees. According to their low mass and vitality, buds of trees in the class of the severest damage (DC4) and in the uppermost crown parts of three out of the four saplings in DC3 did not unfold to leaves in spring. The finding of all these legacy effects on buds upon drought confirmed the existence of the expected interindividual differences among the beech saplings in their susceptibility to severe drought. Intraspecific differences in survival, growth and parameters of photosynthesis among provenances or cultivars subjected to drought stress were also found in seedlings of Scots pine (*Pinus sylvestris*) [34] and grafts of apple trees *(Malus × domestica)* [35]. Our result of a lower bud mass in more severely drought-stressed trees is in accordance with earlier findings of a reduced bud mass in severely drought-stressed seedlings of European beech and pubescent oak (*Quercus pubescens*), whereas in sessile oak (*Q. petraea*) and rowan (*Sorbus aucuparia*), which also can grow at dry sites, only the number, but not the individual mass of the buds was reduced [23]. The reduction in bud number can be interpreted in two ways: solely as a result of reduced carbon gain via photosynthesis during the drought period, or as a consequence of altered carbon allocation towards repair or enhanced formation of water-transport tissue as a prerequisite of a future positive carbon balance [36] at the expense of bud formation. In grafts of apple trees, for instance, drought stress not only resulted in decreases in the size and mass of the leaves, but also in a reduction in vessel area, density and diameter and, consequently, in a decrease in the hydraulic conductance and sap flow [35]. In our study, the findings of significantly reduced numbers and masses of the individual buds could be explained by either of these two mechanisms, but these reductions may simply be due to reduced photosynthesis via lowered stomatal conductance, which results in higher (less negative) δ^13^C ratios. Accordingly, shaded or only weakly illuminated saplings or cuttings of broadleaf North American or Asian tree species exhibited ^13^C enrichment, due to reduced ^13^C discrimination, and lowered bud biomass upon a decrease in stomatal conductance compared to non-shaded or well-illuminated plants [37,38]. In addition, prolonged drought can also exert an immediate impact on the biochemistry of photosynthesis by impairing photosystem II with negative effects on growth and survival, as has been found in several provenances of the Scots pine [34].

Our second hypothesis was only partly confirmed, as there were no significant differences in leaf mass, leaf size and SLA among the damage classes, and thus, no legacy effects of drought on leaf morphology. However, we found significantly increased foliar δ^13^C ratios in at least one of the damage classes and a significantly positive correlation between bud and leaf δ^13^C. This finding suggests that the unfolded leaves were generated under consumption of ^13^C-enriched NSC synthesised under drought stress in the preceding year. Increased δ^13^C ratios in foliar NSC upon drought have also been found in mature European beech trees and in saplings of European oak species (pedunculate oak, *Quercus robur*; and sessile oak) [26,39]. In general, foliar δ^13^C also is a suitable proxy of plant water relations and water-use efficiency in seedlings of trees depending on the duration and intensity of drought, the time of sampling and precipitation events (e.g., [40] and references therein). The fact that in our study, foliar δ^13^C was increased in only one of the damage classes (DC2), the lack in significant differences among the damage classes in leaf morphology (mass, size, SLA) and the non-significant correlations between bud mass and leaf morphology might, at least in part, be due to a delay in bud burst of up to two years after bud formation, which has been observed in mature European beech trees [41]. However, this is not very probable for very young beech trees that need to rely on rapid growth and would not be in line with the above-stated correlation between bud and foliar δ^13^C. 

As is typical of the European beech, as a tree species that generates characteristic sun and shade leaves with all intermediate forms, the SLA decreased from the lower to the upper crown part, implying that towards the crown top, the leaves become smaller but thicker. As could be expected from the early developmental stage of the saplings compared to mature trees, their SLA rather was typical of shade leaves of adult specimens (≥20 m^2^ kg^−1^) than of sun leaves, which generally attain values of 8–16 m^2^ kg^−1^ [42,43,44,45].

As we had put forward in our third hypothesis, bud δ^13^C was also significantly correlated with the bud mass and, according to the significant correlation between bud mass and vitality, also with the vitality of the buds. The explanatory power of the ordinal regressions on vitality is somewhat restricted, as only three classes of bud vitality could be considered, but the clear trends are nevertheless obvious from the scatter plots shown in Figure 2a,c, and the concordance among the relationships among the vitality, mass and δ^13^C of the buds confirms our conclusion. 

In contradiction to our fourth hypothesis, bud mass and leaf morphology were not significantly related to each other. This finding might be due to a certain extent of resilience of the European beech after drought stress, which was found in several previous studies. Beech seedlings were able to regain a high relative water content upon rewatering after severe drought stress, probably because they retained a relatively low (more negative) leaf water potential [23]. Recovery of the trees should also be facilitated by the observed fast recuperation of gas exchange and tree-ring growth [46]. A recovery in the extent of foliation and leaf colouration, as well as in the basal area increment of the stems, was also detected in mature beech trees in the year following a year with a severe drought in spring and summer [3]. Recreation in beech saplings was also observed in the understorey beneath the canopy of forest stands in the year after a year of severe summer drought [47]. In our study, all trees of the moderate damage classes (DC1 and DC2) leaved out completely in spring after the year of drought stress, but no leaves were formed in the uppermost crown part of DC3 trees and no leaf formation occurred in DC4 plants, which shows interindividual differences due to various extents of plasticity. Leaf unfolding in the damaged trees might have been supported by a relatively large N pool in the plants. This can be concluded from the finding that the foliar N concentrations in the trees of the remaining classes with the severest damage (DC2 and DC3) were significantly higher than in the control trees, whose N concentrations were still in the normal range for beech seedlings and saplings of approximately 2.0–2.3%, whereas the N concentrations in the trees of DC2 and DC3 were already in the optimum range [48,49]. Efficient N resorption from senescing leaves of drought-stressed woody plants has been found in deciduous species of eastern North America [50]. A reduced biomass formation in the spring after drought may also have contributed to the higher foliar N concentrations of the trees in DC2 and DC3. 

From all those results, it can be concluded that the European beech is not resistant, but resilient to a certain extent, at least to a singular drought stress [46]. At the species level, the resilience of the European beech to drought may be due to a relatively high plasticity in its hydraulic features not only at the individual level within a given population [51]. Significant differences in the individuals’ drought-related xylem vulnerability [52,53] and also in the production and partitioning of biomass [54], soil-water exploitation, leaf water potential, transpiration, foliar δ^13^C, leaf morphology and foliar NSC [55,56,57,58] have also been detected among different populations. However, the capability of the European beech to recover from drought stress also seems to depend on the local climatic and edaphic conditions [8]. Nevertheless, the species’ plasticity within and among population opens up options for selecting suitable provenances for rejuvenation and afforestation, e.g., within the framework of assisted migration [59,60]. However, the species’ resilience may be substantially diminished when periods of severe drought stress occur in successive years [61]; this occurred in many regions of Central Europe in 2018–2020, followed by another hot and dry summer in 2022. 

## 5. Conclusions

For the first time, we have combined assessments of the vitality, mass and δ^13^C ratios of buds from severely drought-stressed woody plants with morphological and physiological variables of leaves that have emerged from the same plants and crown parts. Bud vitality and δ^13^C have proven to be suitable indicators of the extent of drought-induced injury to plants. Bud vitality may be used as a simple means of screening saplings for the flushing capability in the subsequent spring. The isotopic signature of the buds was still imprinted on the leaves emerged in the following spring. In contrast, the lack of significant differences in morphological leaf variables among the drought damage classes in the subsequent spring is indicative of a relatively high resilience of the beech to drought stress. Differences among individuals and populations in the susceptibility of the beeches to drought are due to a high plasticity in traits related to water relations and biomass partitioning. They provide a basis for selecting suitable provenances for rejuvenation and afforestation. Such options, however, may be restricted by an increased frequency of hot and dry summer periods, which are projected in the future course of climate change.

## Figures and Tables

**Table 1:** Numbers, morphological and physiological variables of young beech trees and buds in different tree damage (DC) and bud vitality classes after a dry summer (mean values ± 1 SE, where applicable). Capital letters refer to statistical comparisons among damage classes, lower-case letters indicate statistically different values among crown regions. Values marked with the same letter are not significantly different.

Variable	DC1	DC2	DC3	DC4
*Total number of trees*	*7*	*5*	*14*	*14*
Tree height (cm)	125.8 ± 3.8	126.2 ± 6.6	127.8 ± 2.6	134.6 ± 3.9
Stem diameter (mm)	15.3 ± 0.7	14.4 ± 0.6	14.1 ± 0.3	14.7 ± 0.4
Extent of damage to the stem (%)	1 ± 1	6 ± 2	20 ± 5	60 ± 7
Extent of damage to the leaves (%)	9 ± 4	44 ± 10	83 ± 3	96 ± 1
Number of buds	208 ± 22 **A**	121 ± 17 **AB**	104 ± 14 **B**	81 ± 15 **B**
*Number of trees for physiological analyses*	*4*	*4*	*4*	*4*
Individual bud fresh mass, upper crown (mg) ^1)^	41.6 ± 3.1	16.4 ± 3.7	5.4 ± 0.7 **b**	7.3 ± 1.0
Individual bud fresh mass, middle crown (mg) ^1)^	40.7 ± 2.7	31.1 ± 5.4	15.7 ± 2.9 **a**	9.5 ± 2.5
Individual bud fresh mass, lower crown (mg) ^1)^	42.7 ± 5.0	27.6 ± 4.3	18.4 ± 2.9 **a**	9.1 ± 2.4
Individual bud fresh mass, entire crown (mg)	41.7 ± 2.2 **A**	25.0 ± 2.9 **B**	13.2 ± 1.8 **C**	8.6 ± 1.2 **C**
Bud number per bud vitality class, upper crown ^2)^	7 / 1 / 0	4 / 1 / 3	0 / 0 / 8	0 / 0 / 8
Bud number per bud vitality class, middle crown ^2)^	8 / 0 / 0	8 / 0 / 0	2 / 2 / 4	0 / 0 / 8
Bud number per bud vitality class, lower crown ^2)^	6 / 2 / 0	8 / 0 / 0	4 / 2 / 2	2 / 2 / 4
δ^13^C, upper crown (‰) ^1)^	−27.8 ± 0.4 **B**	−25.8 ± 0.3 **A**	−24.7 ± 0.2 **Aa**	−24.9 ± 0.9 **A**
δ^13^C, middle crown (‰) ^1)^	−27.2 ± 0.3	−26.9 ± 0.6	−25.3 ± 0.3 **a**	−25.2 ± 0.4
δ^13^C, lower crown (‰) ^1)^	−27.5 ± 0.2	−27.2 ± 0.5	−28.0 ± 0.5 **b**	−26.6 ± 1.1
δ^13^C, entire crown (‰)	−27.5 ± 0.2 **B**	−26.6 ± 0.3 **AB**	−26.0 ± 0.4 **A**	−25.6 ± 0.5 **A**

^1)^ Two buds per tree and crown part resulting in eight buds per damage class and crown position; ^2)^ bud vitality classes according to TTC tests in the sequence “high vitality”/“reduced vitality”/“sub-vital”; maximum bud number per damage class and crown position = 8 (two buds per tree and crown position).

**Table 2:** Results of Two-Way ANOVA applied to variables of buds (harvested in autumn 2020) and leaves (sampled in spring 2021) of different damage classes and crown parts of European beech saplings subjected to severe drought stress in spring and summer 2020. Significant results are printed in bold. n.t.—not tested due to unbalanced design (missing data in the upper crown part).

Variable	Damage Class			Crown Part			Damage Class × Crown Part		
	*df*	*F*	*p*	*df*	*F*	*p*	*df*	*F*	*p*
Individual bud fresh mass	3	**51.155**	**<0.001**	2	**4.647**	**0.012**	6	1.283	0.274
Bud δ^13^C	3	**7.436**	**<0.001**	2	**9.272**	**<0.001**	6	**2.418**	**0.033**
Individual leaf dry mass	3	0.629	0.601	2	0.829	0.445	6	0.919	0.494
Leaf size	3	0.251	0.860	2	0.058	0.944	6	0.564	0.756
SLA	3	2.062	0.124	2	**18.401**	**<0.001**	6	1.555	0.191
Leaf δ^13^C	3	**10.285**	**<0.001**	2	2.667	0.082	n.t.	n.t.	n.t.
Foliar N	3	**21.626**	**<0.001**	2	0.130	0.878	n.t.	n.t.	n.t.

**Table 3:** Leaf features of European beech saplings (5 leaves per tree and crown part) of different damage classes (DC) in spring after a year of severe drought stress (mean values ± 1 SE). Capital letters refer to statistical comparisons among DC; lower-case letters indicate significant differences among crown regions. Values marked with the same letter are not significantly different. n.d.—no data available.

Variable	Control	DC1	DC2	DC3
*Number of trees*	*4*	*4*	*4*	*4*
Individual leaf dry mass, upper crown (mg) ^1)^	62.7 ± 8.2	65.6 ± 11.5	50.4 ± 8.6	32.7 ^1)^
Individual leaf dry mass, middle crown (mg) ^1)^	39.8 ± 4.5	48.2 ± 8.0	40.0 ± 10.7	59.6 ± 14.3
Individual leaf dry mass, lower crown (mg) ^1)^	36.4 ± 3.0	49.3 ± 6.2	46.0 ± 6.4	38.1 ± 4.9
Individual leaf dry mass, entire crown (mg)	46.3 ± 4.7	54.3 ± 5.6	45.5 ± 5.2	47.0 ± 7.7
Leaf size, upper crown (cm^2^)	11.7 ± 1.3	12.5 ± 1.7	9.9 ± 1.5	7.4 ^1)^
Leaf size, middle crown (cm^2^)	10.0 ± 1.1	10.8 ± 1.5	9.6 ± 2.5	13.2 ± 2.9
Leaf size, lower crown (cm^2^)	10.1 ± 0.7	11.4 ± 1.2	11.4 ± 1.8	10.5 ± 1.4
Leaf size, entire crown (cm^2^)	10.6 ± 0.6	11.6 ± 0.9	10.3 ± 1.2	11.3 ± 1.6
SLA, upper crown (m^2^ kg^−1^)	18.9 ± 0.6 **b**	20.0 ± 0.9	20.4 ± 0.7 **b**	22.9 ^1)^
SLA, middle crown (m^2^ kg^−1^)	25.7 ± 1.4 **a**	23.3 ± 1.5	24.6 ± 0.4 **a**	23.8 ± 1.1
SLA, lower crown (m^2^ kg^−1^)	28.2 ± 0.9 **a**	24.0 ± 1.3	25.2 ± 0.8 **a**	27.6 ± 0.7
SLA, entire crown (m^2^ kg^−1^)	24.3 ± 1.3	22.4 ± 0.9	23.4 ± 0.7	25.4 ± 0.9
δ^13^C, upper crown (‰)	−26.0 ± 0.4	−26.4 ± 0.4	−24.4 ± 0.5	n.d.
δ^13^C, middle crown (‰)	−26.2 ± 0.5	−26.6 ± 0.3	−24.8 ± 0.3	−25.8 ± 0.3
δ^13^C, lower crown (‰)	−26.5 ± 0.5	−27.0 ± 0.3	−25.4 ± 0.2	−26.4 ± 0.4
δ^13^C, entire crown (‰)	−26.2 ± 0.5 **B**	−26.7 ± 0.3 **B**	−24.8 ± 0.3 **A**	−26.1 ± 0.3 **B**
N, upper crown (%)	2.26 ± 0.03	2.27 ± 0.14	2.87 ± 0.04	2.97 ^1)^
N, middle crown (%)	2.27 ± 0.03	2.30 ± 0.15	2.80 ± 0.07	2.86 ± 0.04
N, lower crown (%)	2.52 ± 0.01	2.21 ± 0.15	2.77 ± 0.08	2.87 ± 0.07
N, entire crown (‰)	2.35 ± 0.05 **B**	2.26 ± 0.09 **B**	2.81 ± 0.04 **A**	2.87 ± 0.04 **A**

^1)^ Only one value.

## Data Availability

Data available on request from the corresponding author.

## Supplementary Materials

The following supporting information can be downloaded at: https://www.mdpi.com/article/10.3390/plants12030568/s1, Figure S1. Buds of the European beech (*Fagus sylvatica* L.) stained with TTC (2,3,5-triphenyltetrazolium chlo-ride) as a vitality test. (a) High vitality and flushing capability; (b) reduced vitality; (c) sub-vital. See the main text for further explanation.
