# Supplementary material for: Legacy Effects in Buds and Leaves of European Beech Saplings (Fagus sylvatica) after Severe Drought
Source: Plants (Basel). 2023 Jan 26;12(3):568. doi: 10.3390/plants12030568 (PMC9920899; doi:10.3390/plants12030568)
Supplement: Supplementary file 1 [file plants-12-00568-s001.zip › plants-2005006-supplementary.pdf]

Supplementary Material to:

Legacy Effects in Buds and Leaves of European Beech Saplings (*Fagus sylvatica*) after Severe Drought

Frank M. Thomas *, Lena Schunck and Alexis Zisakos

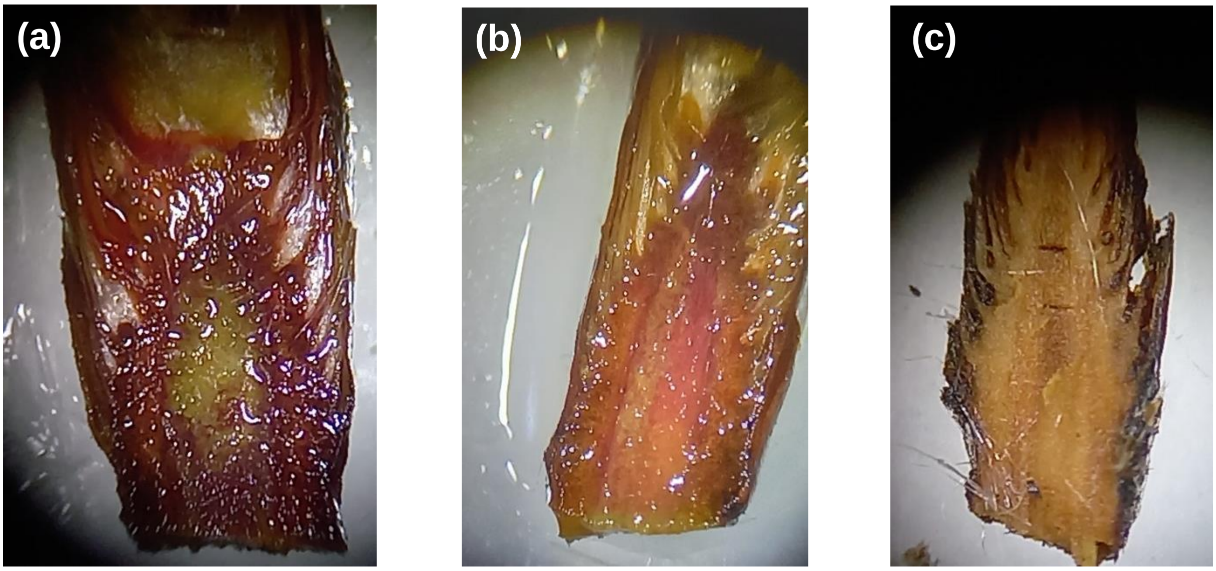

Figure S1. Buds of the European beech (*Fagus sylvatica* L.) stained with TTC (2,3,5-triphenyltetrazolium chloride) as a vitality test. **(a)** High vitality and flushing capability; **(b)** reduced vitality; **(c)** sub-vital. See the main text for further explanation.
